# Orthodontic Treatment as a Protective Factor for Dental Caries Experience and Severity: A Population-Based Study

**DOI:** 10.1155/2021/9926069

**Published:** 2021-04-28

**Authors:** Ahmed A. Alsulaiman

**Affiliations:** Department of Preventive Dental Sciences, Imam Abdulrahman Bin Faisal University, College of Dentistry, Dammam, Saudi Arabia

## Abstract

**Objective:**

The aim of this study was to assess the effects of orthodontic treatment on the experience, prevalence, and severity of dental caries later in life in a representative sample of U.S. adults.

**Methods:**

Using a population-based study design, data from 9,486 participants in the third National Health and Nutrition Survey (NHANES), including self-reported information on the history of orthodontic treatment and its timing, were obtained. Caries experience and prevalence was assessed using the decayed (DT) and filled (FT) teeth indices (i.e., DT > 0, FT > 0, and their aggregate DFT > 0). Severe dental caries experience and prevalence was defined as DT > 2, FT > 11, and DFT > 12. Unadjusted and adjusted logistic regression models that accounted for the complex sampling design were used to assess the association between orthodontic treatment and dental caries experience, prevalence, and severity. Statistical significance was set at a *P* value of less than 0.05.

**Results:**

The history of orthodontic treatment was reported in 19.62% of the adults. Around 94% of participants had at least one decayed or filled tooth (DFT > 0), and 21.09% met the aggregate DFT criterion for severe caries (DFT > 12). After controlling for confounding variables, a reported history of orthodontic treatment was found to significantly decrease the odds of DT > 0, DT > 2, FT > 11, and DFT > 12 (odds ratios (OR) = 0.41, 0.36, 0.74, and 0.60, respectively).

**Conclusion:**

A history of orthodontic treatment was a protective factor for untreated dental caries, in assessments of the severity and prevalence of dental caries experience.

## 1. Introduction

Dental caries is one of the highly prevalent chronic diseases worldwide [1]. In the United States (U.S.), the most recent oral health surveillance report indicated that more than 90% of adults had experienced dental caries in their lifetime, and approximately 32% of them had untreated decay [2]. Untreated dental caries is responsible for chewing deficiencies, pain, infection, and ultimately, tooth loss [3–5]. Thus, dental caries can severely diminish an individual's normal life activities, work productivity, sleep, and overall quality of life [6]. Moreover, treatment of dental caries is expensive, with the World Health Organization (WHO) reporting dental caries treatment as the fourth most expensive among therapeutic costs for all diseases [7]. Fortunately, dental caries is a preventable disease [8]. Therefore, recognition of individual factors (i.e., risk indicators or factors) that can indicate a person's susceptibility to develop dental caries and management or even prevention of those factors early in life is important [9].

Malocclusion is one of the factors that contribute to the development of dental caries [3, 10–13]. Malocclusion refers to deviations from the ideal occlusion of the teeth and jaws and encompasses distinct components, including tooth-jaw size discrepancies (crowding, irregularity, and spacing) and disharmony of the maxillary and mandibular arches (i.e., in the sagittal, transverse, and vertical planes) [14]. Malocclusion traits can be successfully alleviated by orthodontic treatment [15], which largely aims to enhance the social and psychological well-being of patients by improving their appearance [16]. However, current research is focused on the oral health benefits of orthodontic treatment, beyond its esthetic benefits [17]. Data outlining the ability of orthodontic treatment to reduce the risk of dental caries development later in life remains contradictory and inconclusive [18–21] with low methodological quality [17]. The low quality of these studies can be mainly attributed to the absence of a detailed sampling process, the exclusion of response rate reporting, and inappropriate sample size calculation [17].

To the best of my knowledge, no study has investigated the association between orthodontic treatment and dental caries prevalence while accounting for the severity of caries. Identification of high-risk individuals would allow for the application of the appropriate strategies, wherein those with severe dental caries can benefit from individual-specific prevention approaches such as orthodontic treatment [22]. A recent systematic review [17] indicated an increasing need for additional studies to test the effects of orthodontic treatment on the development of dental caries later in life. Thus, this population-based cross-sectional study aimed to assess the effects of orthodontic treatment on dental caries experience, prevalence, and severity in adults in the U.S.

## 2. Materials and Methods

### 2.1. Survey Description

In the U.S., the National Health and Nutrition Examination Survey (NHANES) is a program managed by the National Center for Health Statistics (NCHS), a part of the Centers for Disease Control and Prevention (CDC) with the objective of obtaining vital health-related statistics in the 50 states representing 81 counties across the U.S. NHANES is a major NCHS program that collects annual health, social, and nutritional information from a nationally representative sample using a cross-sectional design with a complex, stratified, multistage probability cluster sampling process representing the civilian noninstitutionalized population of the U.S. household interviews (consisting of sociodemographic, dietary, and health-related questions) are conducted, and subjects are invited to mobile examination centers (MEC) to complete the health examination component (i.e., medical, dental, physiological, and laboratory examination) [23]. Additional detailed information on the study protocol has been reported previously [24–26]. Informed consent was obtained from all participants. The NHANES study protocols are in accordance with the Declaration of Helsinki and received approval from the National Center for Health Statistics Ethics Review Board [25, 26].

### 2.2. Analytic Sample

For this study, NHANES cycles that included both information on the history of orthodontic treatment and a complete examination of the dental caries component were needed; therefore, NHANES III (i.e., the third series of NHANES surveys) data collected from 1988 to 1994 (i.e., 6 years data) were used in the analysis [27]. The total sample size of NHANES III was approximately 40,000 participants aged 2 months and older representing about 250 million civilians from the noninstitutionalized population [27]. Participants who met the following criteria were included in the study: (1) completed the clinical assessment of coronal caries and (2) provided their responses to the questionnaire component that covered information about the history of previous orthodontic treatment. This yielded an analytical sample of 9,486 participants representing more than 115 million individuals of the U.S. population.

### 2.3. Coronal Caries Assessment

In the oral health room of the MEC, licensed and calibrated dentists utilized a fiberoptic light and a mirror with a No. 23 explorer to conduct oral examinations of the occlusal, lingual, buccal, mesial, and distal surfaces for all teeth except the third molars. Each tooth surface was then coded as sound (*S*), decayed (*D*), filled (*F*), or missing (*M*). Carious tooth surfaces with a restoration were coded as decayed [25, 28, 29]. For this study, tooth-level data (i.e., at least one decayed or filled surface) were used to produce indices for the number of decayed teeth (DT), filled teeth (FT), and decayed and filled teeth (DFT). In this study, because of the oral examination protocol of NHANES, dental caries indicates cavitated (i.e., dentin level) coronal surfaces, not incipient lesions [29]. The *M* component was excluded from the analysis because it was coded as missing owing to the lack of dental caries or periodontal disease.

The distributions of the DT, FT, and DFT indices were highly skewed, widely seen in the dental caries literature [30]. Therefore, the indices were categorized using two methods: (1) no experience vs. any experience (DT > 0, FT > 0, and DFT > 0) and (2) any experience vs. severe experience (DT > 2, FT > 11, and DFT > 12). The cutoff points of severity corresponded to 21% of the cases with a high caries experience [31], ensuring that the definition of this high-risk group did not surpass 25% of the population studied to allow for effective targeted prevention [22].

### 2.4. Reliability of Dental Caries Assessment

Dentists were periodically trained and calibrated by the National Institute of Dental Research (NIDCR) and an expert dental consultant who operated as the “gold standard” examiner. To assess intraexaminer reliability, the five operating dentists at the MEC performed reexaminations for 30 participants 6 weeks apart, which yielded kappa statistics ranging 0.85–1.00. For interexaminer reliability, the kappa statistics for examinations in 20 volunteers who had received oral examination by both the operating dentists and the “gold standard” examiner ranged 0.96–1.00 [32].

### 2.5. Orthodontic Treatment Information

The household adult questionnaire of the third NHANES contained questions on multiple topics, including those related to the orthodontic treatment and its timing. The two close-ended questions were as follows: (1) “Have you ever received orthodontic treatment?” and (2) “How old were you when you started your orthodontic treatment?” [24]. In addition, the age of the participant at the time of the MEC visit was subtracted from the reported orthodontic treatment age to approximate the number of years between the start of orthodontic treatment and the clinical assessment of dental caries. Although this does not accurately capture the interval from the end of orthodontic treatment to the clinical assessment of dental caries, it offers a recall approximation for those who reported a history of orthodontic treatment.

### 2.6. Covariates

Covariates included age, gender, race, education, income (i.e., socioeconomic status), and frequency of dental visits [24]. Because of the skewness in the age variable, it was categorized according to quartiles (young adults: <24 years, adults: ≥24 and <39 years, and older adults: ≥39 years). The reported races and ethnicities in the third NHANES included Caucasian, African-American, Mexican-American, and other races. Education was recorded as the number of educational years the participant had completed. Twelve years of education was used as a cutoff point to categorize participants into those possessing less than high school education or high school graduate or higher. Socioeconomic status (SES) was quantified by the poverty income ratio (PIR), which is the ratio of annual family income to the federal poverty line. SES was categorized as follows: low SES: PIR = 0–1.3, medium SES: PIR = 1.3–3.5, and high SES: PIR > 3.5 [33]. Frequency of dental visits was reclassified as at least one visit annually to the dentist or hygienist or no visits to the dentist or hygienist on a yearly basis.

This study followed the STROBE (i.e., Strengthening the Reporting of Observational studies in Epidemiology) guidelines for cross-sectional studies [34].

### 2.7. Statistical Analysis

To analyze the sampled data, the MEC examination weights and SURVEY procedures in SAS version 9.4 (SAS Institute Inc., Cary, NC, USA) were used to account for the complex sampling design and generate unbiased estimates and variance [35]. The associations among the main outcomes (i.e., DT, FT, and DFT), main predictor (i.e., the history of orthodontic treatment), and other included covariates (i.e., age, gender, race, education, income, and frequency of dental visits) were tested using the chi-square statistical test. Unadjusted and adjusted logistic regression models were used to report odds ratios (OR) and 95% confidence intervals (CI) of the effect of a history of orthodontic treatment on dental caries experience and severity. Covariates that showed a significant association with the outcome variable and main predictor or altered the measure of association of the main predictor by 10% were defined as confounders. Interaction terms between the main predictor (i.e., the history of orthodontic treatment) and the covariates identified as confounders were assessed. The significance level was evaluated at an alpha level of less than 0.05.

## 3. Results

### 3.1. Descriptive Results

Participants were mostly Caucasian (73.14%; SE: 1.42), female (51.41%; standard error (SE): 0.55), and adults (age ≥24 and <39 years: 50.41%; SE: 0.98) with at least a high school degree or higher (79.55%; SE: 1.00) and within the medium SES category (43.13%; SE: 1.30), and all participants reported at least one visit to the dentist yearly (Table 1).

Approximately 20% (SE: 0.96) of the studied population had undergone orthodontic treatment previously. This population included a total sample size of 1125 participants representing approximately 23 million adults who had undergone orthodontic treatment. The mean age when orthodontic treatment started was approximately 16 years (mean: 15.72 years; SE: 0.28), and the mean recall clinical assessment was around 14 years (mean: 13.78 years; SE: 0.37 years). Approximately 28% of the participants had at least one tooth with untreated decay (DT > 0), 87.16% had at least one filled tooth (FT > 0), and 93.48% had at least one decayed or filled tooth (DFT > 0). Around 11% of the participants showed a severe DT index (DT > 2), 22.17% had a severe FT index (FT > 11), and 21.09% had severe DFT index (DFT > 12) (Table 1).

### 3.2. Bivariate Results

Characteristics of the study population by the history of orthodontic treatment are provided in Table 2. In summary, a history of orthodontic treatment was most prevalent among young participants (<24 years), Caucasian females, those with an educational level of high school or more, and those belonging to the high SES group (Table 2).

Characteristics of the study population by caries experience (DT > 0, FT > 0, and DFT > 0 indices) are provided in Table 3. In summary, a significantly higher proportion of DT > 0 cases was observed among participants aged 24 years or older, Caucasian females, individuals within the low SES category, those with less than high school education, and those who reported no history of orthodontic treatment. A significantly higher proportion of FT > 0 cases was observed among those aged 39 years or older, Caucasian females, those within the high SES category, those with high school education or more, and those with a reported history of orthodontic treatment (Table 3). A significantly higher proportion of DFT > 0 cases was observed among those aged 39 years or older, Caucasian females, those within the high SES category, and those with high school education or more (Table 3). In summary, a history of orthodontic treatment significantly decreased the prevalence of untreated dental caries (DT > 0) but significantly increased the prevalence of FT (FT > 0) and had no significant association with the aggregate DFT index (DFT > 0).

Characteristics of the study population by caries experience severity (i.e., the DT > 2, FT > 11, and DFT > 12 indices) are provided in Table 3. A higher prevalence of untreated carious teeth (DT > 2) was observed among participants aged 24–39 years, African-American males, those within the low SES category, those with less than high school education, and those with no history of orthodontic treatment. A higher prevalence of filled teeth (FT > 11) was observed among those aged 39 years or more, Caucasian females, those within the high SES category, those with high school education or more, and those who reported no history of orthodontic treatment (Table 3). A higher prevalence of dental caries (DFT > 12) was observed among those aged 39 years or older, Caucasian females, those within the high SES category, those with high school education or more, and those with no history of the reported orthodontic treatment (Table 3). In summary, a history of orthodontic treatment significantly decreased the prevalence of the DT > 2, FT > 11, and DFT > 12 indices.

### 3.3. Multivariate Analysis

After controlling for the confounders identified in Table 4 (i.e., age, gender, race/ethnicity, SES, and education), those who had previously received orthodontic treatment had an OR of 0.41 (95% CI: 0.33–0.51; *P* value: < 0.0001) for DT > 0 in comparison to those who had not reported previous orthodontic treatment (i.e., the reference group). After controlling for confounders, those who received previous orthodontic treatment had an odds ratio (OR) of 1.67 (95% CI: 1.07–2.61; *P* value: 0.03) for FT > 0 compared to the reference group. Similarly, after controlling for confounders, those who had previously received orthodontic treatment had an OR of 0.97 (95% CI: 0.62–1.50; *P* value: 0.87) for DFT > 0 compared to the reference group. In summary, a history of orthodontic treatment was a significant protective factor against the prevalence of DT > 0, but increased the odds of FT > 0 and had no association with DFT > 0.

After controlling for the confounders identified in Table 4 (i.e., age, gender, race/ethnicity, SES, and education), those who had received previous orthodontic treatment had an OR of 0.36 (95% CI: 0.22–0.58; *P* value: <0.0001) for DT > 2 compared to those who had not reported previous orthodontic treatment (i.e., the reference group). Similarly, those who had received previous orthodontic treatment had an OR of 0.74 (95% CI: 0.60–0.94; *P* value: 0.01) for FT > 11 compared to the reference group. Those who had received previous orthodontic treatment also showed an OR of 0.60 (95% CI: 0.50–0.77; *P* value: <0.0001) for DFT > 12 compared to the reference group. In summary, a history of orthodontic treatment was a significant protective factor for the severity of dental caries prevalence (i.e., the DT > 2, FT > 11, and DFT > 12 indices).

## 4. Discussion

Identification of the factors that contribute to high caries susceptibility will allow clinicians and public health personal to efficiently apply targeted prevention, which has been recognized to be a cost-effective approach [36]. Despite previous efforts by scholars to examine the association between the history of previous orthodontic treatment and caries development later in life [18–21], none of the reported studies accounted for the highly caries-susceptible individuals (i.e., those with DT > 2, FT > 11, and DFT > 12 in this study population). This cross-sectional study intended to fill this gap in the current literature by using population-based data to comprehensively assess the effect of orthodontic treatment on dental caries experience, prevalence, and severity later in life by using a sample representative of U.S. adults. Ideally, such assessments would use a randomized clinical trial or a prospective cohort study design, but that would introduce ethical issues as to why some participants received treatment and others did not [17]. Thus, the current population-based cross-sectional design may be the best option to answer the questions raised in this study.

The main findings of this study are that a previous history of orthodontic treatment was a significant protective factor for the prevalence of DT > 0, but it increased the odds of FT > 0 and had no association with DFT > 0. When considering the severe forms of caries and their prevalence, the results showed that a history of orthodontic treatment was a significant protective factor against the prevalence of severe dental caries, including all components of the DFT index (i.e., DT > 2, FT > 11, and DFT > 12). These results may be explained by the likelihood that malocclusion, which is a risk factor for dental caries, was lesser in those who were orthodontically treated than in those who were not treated [10–13]. Proffit et al. have previously reported that orthodontically treated adults in the NHANES III data had significantly fewer malocclusion traits than those who were not treated [37]. In addition, evidence from the literature shows that those with a history of orthodontic treatment had superior oral healthcare and practice than those without a history of orthodontic treatment [21].

Notably, the FT > 0 and FT > 11 indices show contradicting results, with a history of orthodontic treatment being associated with a significantly increased odds of FT > 0 and a significantly decreased odds of FT > 11. The increased odds of FT > 0 is likely attributable to the fact that those with a history of orthodontic treatment are more prone to receive at least one filling to initiate treatment [38], and the less likely explanation is that incipient caries (i.e., caries that frequently developed after orthodontic treatment) was left untreated until the cavitated lesions required restorative filling [39]. In addition, the decreased odds of FT > 11 can be attributed to the fact that those with a history of orthodontic treatment in the same population (i.e., adults aged 18–50 years) had significantly fewer orthodontic treatment needs (i.e., no to minor malocclusion) [37] and thus less risk for developing cavitated lesions that required restorative filling [10–12].

Published studies that assessed the association between a history of orthodontic treatment and caries experience later in adulthood have been sparse and inconclusive [18–21]. Most of those studies [18–21] had questionable methodological quality attributable to numerous reasons, including convenient sampling with inherent selection bias, insufficient sample sizes leading to false-negative results (i.e., type II error), incorrect statistical procedures such as parametric analyses on nonnormally distributed dependent variables such as DFT components and aggregates, lack of control for confounding factors, and no accountability for dental caries severity. Two studies [20, 21] were in agreement with this study's findings showing a significant protective association between a history of orthodontic treatment and untreated caries (i.e., DT) later in life, whereas one other study [18] found no significant association. Only two studies [18, 20] investigated the effect of a history of orthodontic treatment on the number of FT and found no significant association, which contradicts the present finding of positive association. In addition, four studies [18–21] found no significant association between a history of orthodontic treatment and the aggregate DFT index later in life, which is in agreement with the present study's findings.

Nevertheless, this study had certain limitations. While NHANES III data are one of the most representative national datasets, its design is restricted to civilian noninstitutionalized U.S. participants. Moreover, oral health information and behavior (i.e., brushing frequency, flossing frequency, and use of oral healthcare products) could not be determined from NHANES III data. Information about the history of orthodontic treatment and its timing was obtained from household interview questionnaires, which might introduce self-reporting bias (i.e., social desirability and recall biases). Because of the cross-sectional design of this study, no conclusions about causality between the studied variables could be derived. Detailed malocclusion status, type of orthodontic treatment (fixed vs. removable), and its actual duration were not available in the NHANES III data.

Despite these limitations, NHANES III data provided us with a unique opportunity to analyze the association between orthodontic treatment and dental caries experience, prevalence, and severity later in life in a representative sample of U.S. adults. Among the numerous strengths of this study are its robust design and analytical approach, the use of a large sample size, and the generalizability of the findings. Although NHANES III data were collected between 1988 and 1994, this study used a subset of data that represents the increased trend of orthodontic treatment in adults and provided a weighted sample for approximately 23 million orthodontically treated patients [40]. In addition, the reported dental caries experience in this study is similar to those lately published by the CDC [2]. Collectively, this demonstrates the distinctive value of the data presented herein in answering the urgent need to determine the effect of orthodontic treatment on dental caries later in life [17].

## 5. Conclusion

The history of orthodontic treatment was a protective factor against untreated dental caries (DT > 0) and the severity of dental caries experience (DT > 2, FT > 11, and DFT > 12) in U.S. adults. This study supports the notion that orthodontically treated patients show improved oral health later in life compared to those who have not received orthodontic treatment. Clinicians and public health administrators should form policies to provide those at high risk of dental caries with appropriate prevention strategies, such as orthodontic treatment, earlier in life.

## Figures and Tables

**Table 1:** Characteristics of the study participants (*n* = 9486).

Characteristics	% (SE)
Age (%)^*∗*^	
<24 y (*n* = 2241)	20.57 (0.76)
≥24 y and <39 y (*n* = 4701)	50.41 (0.98)
≥39 y (*n* = 2544)	29.02 (0.90)

Gender (%)^*∗*^	
Male (*n* = 4262)	48.59 (0.55)
Female (*n* = 5224)	51.41 (0.55)

Race/ethnicity (%)^*∗*^	
Non-Hispanic Caucasian (*n* = 2927)	73.14 (1.42)
Non-Hispanic African-American (*n* = 3204)	12.42 (0.75)
Mexican-American (*n* = 2940)	5.98 (0.52)
Others (*n* = 415)	8.46 (0.94)

Socioeconomic status (%)^*∗*^	
Low SES: 0–1. 3 PIR (*n* = 3004)	17.82 (1.05)
Medium SES: >1.3–3.5 PIR (*n* = 3857)	43.13 (1.30)
High SES: >3.5 PIR and above (*n* = 2625)	39.05 (1.45)

Education level (%)^*∗*^	
Less than high school (*n* = 3105)	20.45 (1.00)
High school or more (*n* = 6381)	79.55 (1.00)

Previous orthodontic treatment (%)^*∗*^	
Yes (*n* = 1125)	19.62 (0.96)
No (*n* = 8361)	80.38 (0.96)

Decayed caries experience (%)^*∗*^	
DT = 0 (*n* = 5878)	71.93 (1.08)
DT > 0 (*n* = 3608)	28.07 (1.08)

Filled caries experience (%)^*∗*^	
FT = 0 (*n* = 2187)	12.84 (0.63)
FT > 0 (*n* = 7299)	87.16 (0.63)

Decayed and filled caries experience (%)^*∗*^	
DFT = 0 (*n* = 933)	6.52 (0.41)
DFT > 0 (*n* = 8553)	93.48 (0.41)

Severe decayed caries experience (%)^*∗*^	
DT ≤ 2 (*n* = 8049)	89.26 (0.59)
DT > 2 (*n* = 1437)	10.74 (0.59)

Severe filled caries experience (%)^*∗*^	
FT ≤ 11 (*n* = 8011)	77.83 (1.21)
FT > 11 (*n* = 1475)	22.17 (1.21)

Severe decayed and filled caries experience (%)^*∗*^	
DFT ≤ 12 (*n* = 8101)	78.91 (1.11)
DFT > 12 (*n* = 1385)	21.09 (1.11)

SE, standard error. ^*∗*^Significant *P* value at *α* < 0.05.

**Table 2:** Characteristics of the study participants who received previous orthodontic treatment (*n* = 9486).

Characteristics	History of orthodontic treatment (*n* = 1125), % (SE)
Age (%)^*∗*^	
<24 y (*n* = 2241)	30.35 (2.10)
≥24 y and <39 y (*n* = 4701)	19.42 (1.35)
≥39 y (*n* = 2544)	12.37 (0.98)

Gender (%)^*∗*^	
Male (*n* = 4262)	15.83 (1.21)
Female (*n* = 5224)	23.21 (1.17)

Race/ethnicity (%)^*∗*^	
Non-Hispanic Caucasian (*n* = 2927)	23.97 (1.15)
Non-Hispanic African-American (*n* = 3204)	5.18 (0.45)
Mexican-American (*n* = 2940)	7.84 (0.55)
Others (*n* = 415)	11.59 (2.39)

Socioeconomic status (%)^*∗*^	
Low SES: 0–1.3 PIR (*n* = 3004)	12.78 (1.45)
Medium SES: >1.3–3.5 PIR (*n* = 3857)	17.58 (1.11)
High SES: >3.5 PIR and above (*n* = 2625)	25.00 (1.38)

Education level (%)^*∗*^	
Less than high school (*n* = 3105)	9.65 (1.34)
High school or more (*n* = 6381)	22.18 (1.01)

SE, standard error. ^*∗*^Significant *P* value at *α* < 0.05.

**Table 3:** Characteristics of study participants with caries experience (*n* = 9486).

Characteristics	Caries experience					
	Any experience			Severe experience		
	DT > 0 (*n* = 3608), % (SE)	FT > 0 (*n* = 7299), % (SE)	DFT > 0 (*n* = 8553), % (SE)	DT > 2 (*n* = 1437), % (SE)	FT > 11 (*n* = 1475), % (SE)	DFT > 12 (*n* = 1385), % (SE)
Age (%)						
<24 y (*n* = 2241)	84.80 (1.25)	75.86 (1.47)	84.80 (1.25)	9.51 (0.96)	4.70 (0.76)	5.63 (0.82)
≥24 y and <39 y (*n* = 4701)	95.33 (0.52)	89.01 (0.87)	95.33 (0.52)	12.36 (0.79)	19.62 (1.42)	18.92 (1.38)
≥39 y (*n* = 2544)	96.41 (0.48)^*∗*^	91.97 (0.75)^*∗*^	96.41 (0.48)^*∗*^	8.79 (0.84)^*∗*^	38.97 (1.90)^*∗*^	35.81 (2.02)^*∗*^

Gender (%)						
Male (*n* = 4262)	92.40 (0.72)	85.07 (1.01)	92.40 (0.72)	12.22 (0.85)	19.43 (1.36)	18.59 (1.28)
Female (*n* = 5224)	94.50 (0.34)^*∗*^	89.14 (0.57)^*∗*^	94.50 (0.34)^*∗*^	9.34 (0.68)^*∗*^	24.75 (1.32)^*∗*^	23.45 (1.22)^*∗*^

Race/ethnicity (%)						
Non-Hispanic Caucasian (*n* = 2927)	94.83 (0.45)	91.46 (0.59)	94.84 (0.45)	8.96 (0.66)	25.68 (1.44)	24.53 (1.34)
Non-Hispanic African-American (*n* = 3204)	88.90 (0.55)	70.70 (1.50)	88.90 (0.55)	20.38 (1.02)	9.89 (0.55)	9.80 (0.48)
Mexican-American (*n* = 2940)	87.40 (0.83)	71.22 (1.31)	87.40 (0.82)	14.79 (0.91)	13.01 (0.68)	11.06 (0.81)
Others (*n* = 415)	92.74 (1.43)^*∗*^	85.48 (2.53)^*∗*^	92.74 (1.43)^*∗*^	9.16 (2.29)^*∗*^	16.27 (2.05)^*∗*^	15.00 (2.05)^*∗*^

Socioeconomic status (%)						
Low SES: 0–1.3 PIR (*n* = 3004)	47.53 (2.09)	74.83 (1.26)	90.39 (0.84)	21.07 (1.43)	10.17 (1.26)	13.61 (1.35)
Medium SES: >1.3–3.5 PIR (*n* = 3857)	29.91 (1.39)	87.32 (0.87)	93.06 (0.60)	11.26 (0.73)	20.26 (1.17)	18.68 (1.21)
High SES: >3.5 PIR and above (*n* = 2625)	17.16 (1.00)^*∗*^	92.61 (0.70)^*∗*^	95.35 (0.53)^*∗*^	5.45 (0.58)^*∗*^	29.75 (1.83)^*∗*^	27.16 (1.73)^*∗*^

Education level (%)						
Less than high school (*n* = 3105)	47.74 (1.62)	72.98 (1.23)	88.56 (0.73)	21.59 (1.13)	8.60 (1.17)	11.14 (0.86)
High school or more (*n* = 6381)	23.01 (1.15)^*∗*^	90.81 (0.55)^*∗*^	94.74 (0.44)^*∗*^	7.95 (0.62)^*∗*^	25.66 (1.40)^*∗*^	23.65 (1.30)^*∗*^

Previous orthodontic treatment (%)						
Yes (*n* = 1125)	11.93 (1.19)	93.06 (1.18)	93.60 (1.17)	3.41 (0.77)	18.90 (1.66)	15.56 (1.53)
No (*n* = 8361)	32.01 (1.15)^*∗*^	85.72 (0.80)^*∗*^	93.45 (0.43)	12.53 (0.68)^*∗*^	22.97 (1.36)^*∗*^	22.44 (1.24)^*∗*^

SE, standard error. ^*∗*^Significant *P* value at *α* < 0.05.

**Table 4:** Crude and adjusted logistic regression models of the association between previous orthodontic treatment and caries experience (*n* = 9486).

Outcome	Main predictor	Crude model	*P* value	Adjusted model^*∗*^	*P* value
		Odds ratio (95% CI)		Odds ratio (95% CI)	
Any caries experience					
DT > 0	Previous orthodontic treatment	0.29 (0.23–0.36)	<0.0001	0.41 (0.33–0.51)	<0.0001
	Yes vs. no (ref.)				
FT > 0	Previous orthodontic treatment	2.23 (1.46–3.41)	0.0004	1.67 (1.07–2.61)	0.03
	Yes vs. no (ref.)				
DFT > 0	Previous orthodontic treatment	1.03 (0.67–1.56)	0.91	0.97 (0.62–1.50)	0.87
	Yes vs. no (ref.)				

Severe caries experience					
DT > 2	Previous orthodontic treatment	0.25 (0.15–0.40)	<0.0001	0.36 (0.22–0.58)	<0.0001
	Yes vs. no (ref.)				
FT > 11	Previous orthodontic treatment	0.78 (0.62–0.98)	0.03	0.74 (0.60–0.94)	0.01
	Yes vs. no (ref.)				
DFT > 12	Previous orthodontic treatment	0.64 (0.50–0.81)	0.001	0.60 (0.50–0.77)	<0.0001
	Yes vs. no (ref.)				

CI, confidence interval. ^*∗*^Adjusted for confounding variables (age, gender, race/ethnicity, socioeconomic status, and education).

## Data Availability

The datasets from the third National Health and Nutrition Examination Survey (NHANES III) used to support the findings of this study are available at https://wwwn.cdc.gov/nchs/nhanes/nhanes3/default.aspx.

## References

[1] Kassebaum N. J., Smith A. G. C., Bernabé E. (2017). Global, regional, and national prevalence, incidence, and disability-adjusted life years for oral conditions for 195 countries, 1990–2015: a systematic analysis for the global burden of diseases, injuries, and risk factors. *Journal of Dental Research*.

[2] Centers for Disease Control and Prevention (2019). *Oral Health Surveillance Report*.

[3] Selwitz R. H., Ismail A. I., Pitts N. B. (2007). Dental caries. *The Lancet*.

[4] Decerle N., Nicolas E., Hennequin M. (2013). Chewing deficiencies in adults with multiple untreated carious lesions. *Caries Research*.

[5] Burt B. A., Eklund S. A. (2005). *Dentistry, Dental Practice, and the Community*.

[6] U.S. Department of Health and Human Services (2000). *Oral Health of America: A Report of the Surgeon General*.

[7] Hosseinpoor A. R., Itani L., Petersen P. E. (2012). Socio-economic inequality in oral healthcare coverage. *Journal of Dental Research*.

[8] Balakrishnan M., Simmonds R. S., Tagg J. R. (2000). Dental caries is a preventable infectious disease. *Australian Dental Journal*.

[9] Fontana M., Zero D. T. (2006). Assessing patients’ caries risk. *The Journal of the American Dental Association*.

[10] Helm S., Petersen P. E. (1989). Causal relation between malocclusion and caries. *Acta Odontologica Scandinavica*.

[11] Singh A., Purohit B., Sequeira P., Acharya S, Bhat M (2011). Malocclusion and orthodontic treatment need measured by the dental aesthetic index and its association with dental caries in Indian schoolchildren. *Community Dental Health*.

[12] Feldens C. A., Dos Santos Dullius A. I., Kramer P. F., Scapini A., Busato A. L. S., Vargas-Ferreira F. (2015). Impact of malocclusion and dentofacial anomalies on the prevalence and severity of dental caries among adolescents. *The Angle Orthodontist*.

[13] Sá-Pinto A. C., Rego T. M., Marques L. S., Martins C. C., Ramos-Jorge M. L., Ramos-Jorge J. (2018). Association between malocclusion and dental caries in adolescents: a systematic review and meta-analysis. *European Archives of Paediatric Dentistry*.

[14] Proffit W. R., Sarver D. M. (2014). *Contemporary Orthodontics*.

[15] Fleming P. S., Seehra J., Polychronopoulou A., Fedorowicz Z., Pandis N. (2013). Cochrane and non-Cochrane systematic reviews in leading orthodontic journals: a quality paradigm?. *The European Journal of Orthodontics*.

[16] Pine C., Harris R. (2007). *Community Oral Health*.

[17] Macey R., Thiruvenkatachari B., O’Brien K., Batista K. B. S. L. (2020). Do malocclusion and orthodontic treatment impact oral health? a systematic review and meta-analysis. *American Journal of Orthodontics and Dentofacial Orthopedics*.

[18] Doğramacı E. J., Brennan D. S. (2019). The influence of orthodontic treatment on dental caries: an Australian cohort study. *Community Dentistry and Oral Epidemiology*.

[19] Thomson W. M. (2002). Orthodontic treatment outcomes in the long term: findings from a longitudinal study of New Zealanders. *The Angle Orthodontist*.

[20] Southard T. E., Cohen M. E., Rails S. A., Rouse L. A. (1986). Effects of fixed-appliance orthodontic treatment on DMF indices. *American Journal of Orthodontics and Dentofacial Orthopedics*.

[21] Choi Y. Y. (2020). Relationship between orthodontic treatment and dental caries: results from a national survey. *International Dental Journal*.

[22] Hausen H. (1997). Caries prediction - state of the art. *Community Dentistry and Oral Epidemiology*.

[23] Centers for Disease Control and Prevention (2020). *NHANES - about the National Health and Nutrition Examination Survey*.

[24] Centers for Disease Control and Prevention (2020). *NHANES Questionnaires, Datasets, and Related Documentation*.

[25] Drury T. F., Winn D. M., Snowden C. B., Kingman A., Kleinman D. V., Lewis B. (1996). An overview of the oral health component of the 1988-1991 national health and nutrition examination survey (NHANES III-phase 1). *Journal of Dental Research*.

[26] Dye B. A., Li X., Lewis B. G., Iafolla T., Beltran-Aguilar E. D., Eke P. I. (2014). Overview and quality assurance for the oral health component of the national health and nutrition examination survey (NHANES), 2009-2010. *Journal of Public Health Dentistry*.

[27] Centers for Disease Control and Prevention (1994). Plan and operation of the Third National Health and nutrition examination survey, 1988–94. series 1: programs and collection procedures. *Vital Health Stat*.

[28] Kaste L. M., Selwitz R. H., Oldakowski R. J., Brunelle J. A., Winn D. M., Brown L. J. (1996). Coronal caries in the primary and permanent dentition of children and adolescents 1–17 years of age: United States, 1988–1991. *Journal of Dental Research*.

[29] National Institute of Dental Research (U.S.) (1991). *Oral Health Surveys of the National Institute of Dental Research: Diagnostic Criteria and Procedures: Epidemiology and Oral Disease Prevention Program*.

[30] Spencer A. J. (1997). Skewed distributions - new outcome measures. *Community Dentistry and Oral Epidemiology*.

[31] Vanobbergen J., Martens L., Lesaffre E., Bogaerts K., Declerck D. (2001). Assessing risk indicators for dental caries in the primary dentition. *Community Dentistry and Oral Epidemiology*.

[32] Beltrán-Aguilar E. D., Barker L. K., Canto M. T. (2005). Surveillance for dental caries, dental sealants, tooth retention, edentulism, and enamel fluorosis--United States, 1988–1994 and 1999–2002. *MMWR Surveill Summ*.

[33] Yang E. J., Kerver J. M., Park Y. K., Kayitsinga J., Allison D. B., Song W. O. (2003). Carbohydrate intake and biomarkers of glycemic control among US adults: the third national health and nutrition examination survey (NHANES III). *The American Journal of Clinical Nutrition*.

[34] Vandenbroucke J. P., von Elm E., Altman D. G. (2007). Strengthening the reporting of observational studies in epidemiology (STROBE). *Epidemiology*.

[35] NHANES C. (2020). Survey methods and analytic guidelines. https://wwwn.cdc.gov/nchs/nhanes/nhanes3/SurveyMethods.aspx.

[36] Burt B. A. (2005). Concepts of risk in dental public health. *Community Dentistry and Oral Epidemiology*.

[37] Proffit W. R., Fields H. W., Moray L. J. (1998). Prevalence of malocclusion and orthodontic treatment need in the United States: estimates from the NHANES III survey. *International Journal of Adult Orthodontics & Orthognathic Surgery*.

[38] Roberts-Harry D., Sandy J. (2003). Orthodontics. part 4: treatment planning. *British Dental Journal*.

[39] Richter A. E., Arruda A. O., Peters M. C., Sohn W. (2011). Incidence of caries lesions among patients treated with comprehensive orthodontics. *American Journal of Orthodontics and Dentofacial Orthopedics*.

[40] Keim R. G., Gottlieb E. L., Nelson A. H. (2013). JCO orthodontic practice study. part 1: trends. *Journal of Clinical Orthodontics*.

